# Risk factors and time-to-event prediction of brain metastasis in patients with lung adenocarcinoma: a retrospective cohort study

**DOI:** 10.3389/fgene.2026.1931724

**Published:** 2026-10-28

**Authors:** Jing Wang, Yuezhen Shen, Yinfu Xu, Jie Liu, Yuchen Guo

**Affiliations:** 1 Department of Oncology, The Second People’s Hospital of Liaocheng, Linqing City, Shandong Province, China; 2 Department of Neurology, The Second People’s Hospital of Liaocheng, Linqing City, Shandong Province, China; 3 Department of Neurosurgery, The Second People’s Hospital of Liaocheng, Linqing City, Shandong Province, China

**Keywords:** brain metastasis, EGFR mutation, lung adenocarcinoma, nomogram, predictive model, risk factors

## Abstract

**Background:**

Brain metastasis (BM) is a clinically important complication of lung adenocarcinoma (LUAD), yet prediction is complicated by variable follow-up, censoring, treatment effects, and competing mortality. This study evaluated time-dependent BM risk and developed an exploratory prediction model in patients with LUAD without BM at baseline.

**Methods:**

Consecutive patients with pathologically confirmed LUAD treated between 2018 and 2024 were retrospectively screened. Patients entered the cohort at the date of a negative baseline contrast-enhanced brain MRI. Incident BM was analyzed using Cox proportional hazards regression. Candidate predictors were prespecified, collinearity was assessed, and LASSO-penalized Cox regression was used for predictor reduction. Internal validation used 1,000 bootstrap resamples. Competing mortality was evaluated using Fine–Gray analysis, and EGFR-TKI exposure was examined as a time-dependent covariate.

**Results:**

Of 94 screened patients, 66 were included; 26 developed incident BM during a median follow-up of 27.8 months. LASSO retained T classification, N classification, EGFR mutation status, and serum CEA. In the parsimonious Cox model, T3–4 disease, N1–3 involvement, and EGFR mutation were associated with higher BM hazard, while the CEA estimate was less precise. The apparent C-index was 0.781 and the optimism-corrected C-index was 0.742. Time-dependent AUCs were 0.773 at 12 months and 0.758 at 24 months. Competing-risk and treatment-adjusted analyses showed directionally consistent findings.

**Conclusion:**

A Cox-based model integrating T stage, N stage, EGFR status, and CEA showed moderate internally validated performance for estimating 12- and 24-month BM-free probabilities. External validation is required before clinical implementation.

## 1 Introduction

Lung cancer remains a leading cause of cancer-related mortality worldwide, accounting for approximately 2.48 million new cases and 1.82 million deaths in 2022 (Sung et al., 2021). Non-small cell lung cancer (NSCLC) represents approximately 80%–85% of lung cancers, and lung adenocarcinoma (LUAD) is its most common histological subtype (Shi et al., 2016; Siegel et al., 2023). Advances in molecularly targeted therapy and immunotherapy have substantially changed the treatment landscape of LUAD; nevertheless, distant metastatic progression remains a major determinant of morbidity and mortality. The brain is a particularly important metastatic site in NSCLC, and lung cancer represents one of the major primary tumors giving rise to brain metastases (BM) (Achrol et al., 2019). Development of BM is associated with neurological impairment, deterioration in quality of life, treatment complexity, and adverse survival outcomes despite improvements in stereotactic radiotherapy and CNS-active systemic therapy (Achrol et al., 2019; Zhang et al., 2022). Identifying patients at increased risk of subsequent BM after a negative baseline brain evaluation therefore remains clinically relevant for risk stratification and longitudinal surveillance.

Brain metastasis is a multistep biological process involving hematogenous dissemination, interaction with the blood brain barrier, adaptation to the neural microenvironment, and subsequent intracranial colonization (Zhang et al., 2022; Wang et al., 2019). Genomic studies have further demonstrated that brain metastases may evolve separately from their corresponding primary tumors and acquire potentially clinically relevant molecular alterations not detected in the primary lesion (Kang et al., 2020). Among molecular characteristics of NSCLC, epidermal growth factor receptor (EGFR) mutation status has received particular attention. Meta-analyses have shown that EGFR-mutant NSCLC is associated with a higher occurrence of BM than EGFR-wild-type disease (Rybarczyk-Kasiuchnicz et al., 2021; Sperduto et al., 2020). Importantly, studies incorporating longitudinal follow-up and competing mortality have also reported an increased incidence of subsequent BM among patients with EGFR-mutant tumors, suggesting that this association cannot necessarily be explained solely by longer survival among patients receiving EGFR-targeted therapy (Brown et al., 2020). Other clinicopathological factors reported in association with BM include younger age, advanced T or N classification, lymphatic involvement, and elevated serum tumor markers such as carcinoembryonic antigen (CEA), although the magnitude and independence of these associations vary across study populations (Hao and Li, 2023).

Several prediction models for BM have consequently been proposed in NSCLC and LUAD. Previous studies have incorporated combinations of tumor stage, lymph-node involvement, histological characteristics, molecular alterations, and serum biomarkers, and some have developed nomograms for estimating subsequent BM risk (Rangachari et al., 2015; Chen et al., 2019; Dunne et al., 2024). However, important methodological limitations remain relevant when interpreting such models. Some studies have treated BM as a binary outcome despite heterogeneous follow-up duration, while others have focused on highly selected populations such as surgically resected NSCLC or patients with established brain metastases (Rangachari et al., 2015; Chen et al., 2019; Dunne et al., 2024). In addition, prediction performance derived from small retrospective cohorts may be susceptible to overfitting if extensive univariable screening and stepwise variable selection are used without adequate internal validation. These issues are particularly important because BM is intrinsically a time-to-event outcome and death before BM represents a potential competing event. Furthermore, molecular epidemiology differs across populations: EGFR mutations are substantially more frequent in Asian than in European NSCLC populations, with pooled estimates approaching approximately 49% and 13%, respectively (Chinese Society of Clinical Oncology, 2021). Therefore, risk models developed in different clinical or molecular contexts may not be directly transferable without population-specific evaluation and appropriate validation.

Accordingly, the present study evaluated the time-dependent risk of incident BM in a retrospective Chinese LUAD cohort with no evidence of brain metastasis at baseline. We aimed to examine clinically available clinicopathological and molecular characteristics associated with subsequent BM and to develop an exploratory Cox-based prediction model for estimating 12- and 24-month brain-metastasis-free probabilities. Given the limited cohort size and number of BM events, model complexity was deliberately restricted and internal validation was performed using bootstrap resampling. Competing mortality and treatment exposure, particularly EGFR-tyrosine kinase inhibitor use, were additionally examined in sensitivity analyses. The resulting model was intended for exploratory risk stratification rather than direct treatment selection and requires external validation before clinical implementation.

## 2 Materials and methods

### 2.1 Study design and cohort construction

This retrospective cohort study was conducted in the Department of Thoracic Oncology at The Second People’s Hospital of Liaocheng and included patients diagnosed with LUAD between January 2018 and December 2024. All consecutive patients with pathologically confirmed LUAD treated in the Department of Thoracic Oncology between January 2018 and December 2024 were screened through the institutional electronic medical record system. No sampling procedure was applied, and all patients from the source population who satisfied the prespecified eligibility criteria were included in the final analytical cohort.

Patients were eligible if they met all of the following criteria: (1) age ≥18 years; (2) histologically or cytologically confirmed LUAD; (3) contrast-enhanced brain magnetic resonance imaging (MRI) performed as part of the baseline staging assessment within 30 days of LUAD diagnosis, with no evidence of brain metastasis; (4) available baseline clinicopathological information; (5) available molecular testing results, including EGFR mutation status; and (6) at least one documented post-baseline clinical or imaging assessment permitting ascertainment of subsequent brain metastasis or vital status.

Patients were excluded if they had: (1) brain metastasis already present on baseline imaging; (2) a concurrent active malignancy or another malignant tumor diagnosed within the preceding 5 years, except adequately treated non-melanoma skin cancer or carcinoma *in situ*; (3) a primary central nervous system tumor or another intracranial disorder that substantially interfered with interpretation of brain imaging; or (4) insufficient baseline information to establish the prespecified candidate predictors.

Importantly, patients were not excluded solely because their follow-up duration was shorter than 6 months. Such patients contributed follow-up time until their last documented assessment and were censored if brain metastasis had not occurred. For the analysis, synchronous brain metastasis was defined strictly as brain metastasis already present on the baseline contrast-enhanced brain MRI. Patients became eligible for prediction at the date of a negative baseline brain MRI, which was defined as the index date. Any newly detected brain metastasis occurring after this index date, including within the first 3 months, was classified as an incident post-baseline event and retained in the analysis. Therefore, the previously used 3-month cutoff was abandoned and no landmark exclusion was applied.

A participant flow diagram (Figure 1) summarizes cohort construction. Among 94 consecutive patients screened during the study period, 28 were excluded because of brain metastasis detected at baseline (n = 17), incomplete baseline clinicopathological or molecular data (n = 6), a concurrent or recent active malignancy (n = 3), or absence of any post-baseline follow-up information (n = 2). The remaining 66 patients constituted the final analytical cohort.

**FIGURE 1:**
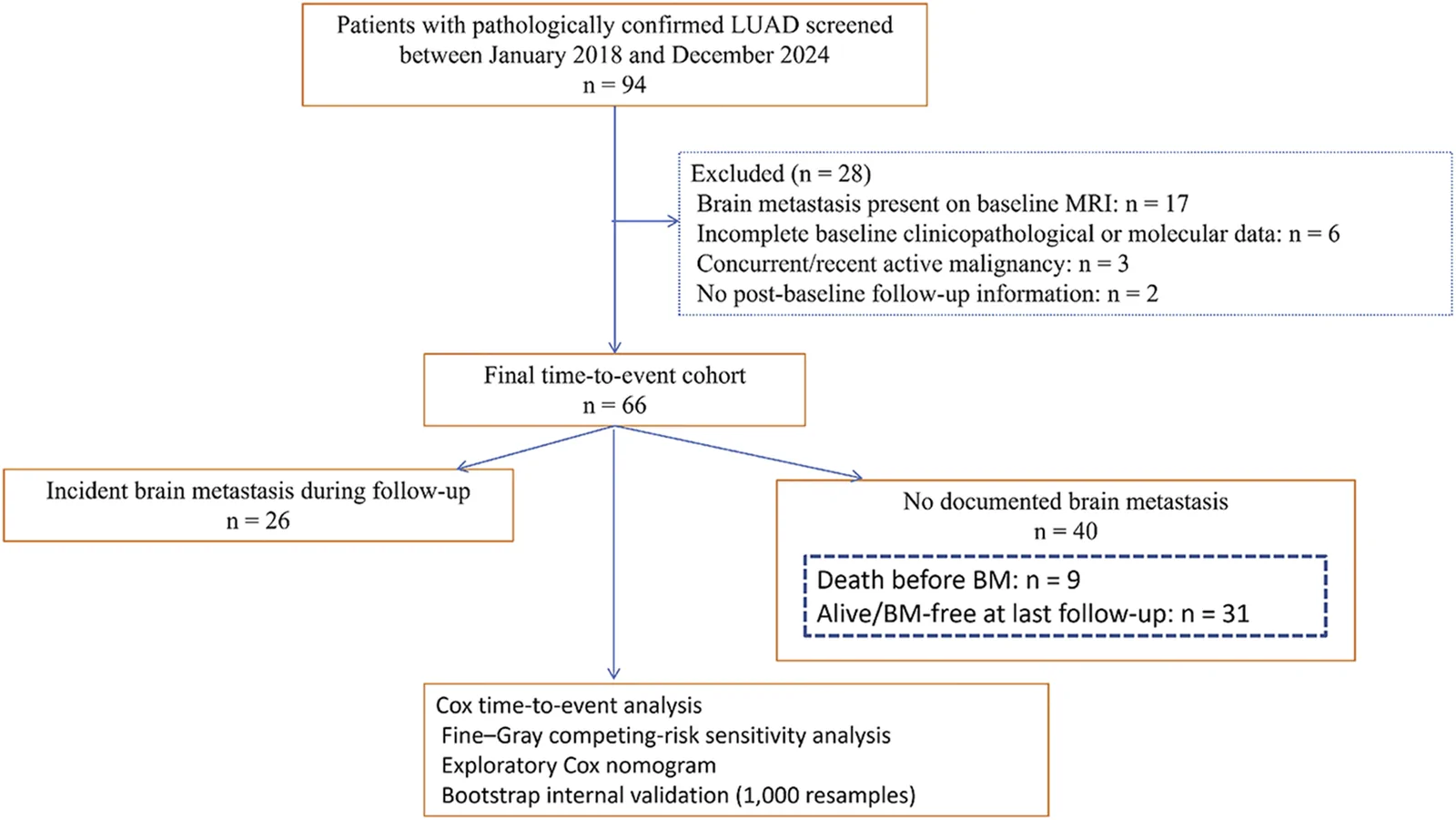
Flow diagram of patient selection, cohort construction, and analytical framework.

Because this was a retrospective study of an available institutional cohort, no formal power-based sample size calculation was used to determine enrollment. All eligible consecutive patients were included. Given the limited number of brain metastasis events, model complexity was deliberately restricted, penalized regression was used for predictor reduction, and the resulting model was considered exploratory.

### 2.2 Index date, follow-up, and outcome definition

The index date (time zero) was defined as the date of the baseline contrast-enhanced brain MRI demonstrating no evidence of brain metastasis. Patients were considered eligible for prediction from this date onward. Brain metastasis present on the baseline MRI was classified as synchronous disease and excluded, whereas any brain metastasis first detected after the index date was classified as incident post-baseline brain metastasis. The baseline MRI was required to have been performed within 30 days of pathological confirmation of LUAD.

The primary outcome was incident brain metastasis during follow-up. Brain metastasis was defined as one or more newly detected intracranial lesions on contrast-enhanced MRI considered radiologically consistent with metastatic disease in the clinical context of LUAD. When additional imaging or multidisciplinary review was performed, the final clinical diagnosis was used for outcome ascertainment. The event date was defined as the date of the first imaging examination confirming brain metastasis.

Brain-metastasis-free survival (BMFS) was defined as the interval from the index date to the first radiologically confirmed brain metastasis. Patients who did not develop brain metastasis were censored at the date of death or their last documented clinical or imaging assessment, whichever occurred first, in the primary cause-specific Cox analysis. Death before brain metastasis was additionally treated as a competing event in the Fine Gray sensitivity analysis. Follow-up duration was calculated from the index date to brain metastasis, death, or the last documented follow-up, as applicable.

### 2.3 Clinical and clinicopathological data collection

Demographic and clinicopathological data were retrospectively extracted from electronic medical records by trained investigators using a standardized data collection form. Baseline demographic variables included age at diagnosis, sex, smoking status, and Eastern Cooperative Oncology Group performance status.

Tumor-related variables included primary tumor location, maximum tumor diameter on computed tomography, T classification, N classification, overall clinical stage according to the eighth edition of the American Joint Committee on Cancer TNM staging system, histological differentiation, and, when available from surgical specimens, visceral pleural and lymphovascular invasion.

Because overall clinical stage is derived substantially from T and N classifications, these variables were considered potentially redundant during prediction modeling. Overall stage was therefore retained for descriptive analysis but was not entered simultaneously with T and N classifications into the final multivariable prediction model.

Baseline serum tumor markers obtained before initiation of systemic anticancer treatment, whenever available, included carcinoembryonic antigen (CEA), cytokeratin 19 fragment (CYFRA21-1), neuron-specific enolase (NSE), and squamous cell carcinoma antigen (SCCA). CEA was categorized using the institutional upper reference limit of 5 ng/mL.

### 2.4 Molecular testing

Tumor tissue obtained from diagnostic biopsy or surgical resection was used for molecular testing. Depending on sample availability, calendar period, and prevailing clinical practice, EGFR alterations were assessed using either next-generation sequencing with a lung cancer-specific gene panel or amplification refractory mutation system polymerase chain reaction.

EGFR status was classified as mutant or wild-type. EGFR-mutant tumors included exon 19 deletions, exon 21 L^85^8 R mutations, and other pathogenic or likely pathogenic EGFR variants identified during routine testing. Individual EGFR mutation subtypes were additionally recorded for exploratory descriptive analyses.

ALK rearrangements were assessed by fluorescence *in situ* hybridization or immunohistochemistry using the Ventana D5F3 assay. KRAS, ROS1, and other driver alterations were recorded when available from routine molecular testing. Because testing panels and testing availability evolved during the 2018–2024 study period, molecular alterations other than EGFR were not required for cohort eligibility and were not used as mandatory candidate predictors in the primary model.

### 2.5 Treatment exposure

Because systemic treatment may influence both survival and central nervous system progression, major anticancer treatments administered after the index date were additionally extracted from the medical records.

Treatment variables included EGFR tyrosine kinase inhibitor (EGFR-TKI) therapy, specific EGFR-TKI generation or agent when available, cytotoxic chemotherapy, immune checkpoint inhibitor therapy, thoracic radiotherapy, and other clinically relevant extracranial radiotherapy. For EGFR-TKI therapy, both treatment initiation date and treatment discontinuation or change were recorded whenever available.

Treatment characteristics were summarized descriptively in the primary cohort. Because EGFR-TKI treatment could be initiated after cohort entry, treatment exposure was not treated simply as a fixed baseline predictor in the primary prediction model. In a prespecified sensitivity analysis, EGFR-TKI use was modeled as a time-dependent exposure, with patients classified as unexposed before treatment initiation and exposed thereafter. This approach was used to reduce potential immortal-time bias associated with treatment initiated during follow-up.

Treatment adjusted analyses were considered exploratory because treatment allocation was not randomized and was influenced by molecular status, disease stage, calendar period, and clinical condition. Calendar period was additionally reviewed descriptively because systemic treatment patterns changed substantially between 2018 and 2024, particularly after broader adoption of third-generation EGFR-TKIs and immune checkpoint inhibitors. Given the limited number of BM events, calendar period was not added as an additional parameter to the primary prediction model in order to avoid further model overfitting.

### 2.6 Brain imaging surveillance

All included patients underwent contrast-enhanced brain MRI during baseline staging. Subsequent brain imaging was performed according to routine clinical practice, generally at scheduled follow-up intervals and additionally when neurological symptoms or other clinical findings raised concern for intracranial progression.

Where available, dates of follow-up brain MRI examinations were extracted to characterize surveillance intensity. The number and interval of brain imaging examinations during follow-up were summarized descriptively to assess whether substantial differences in surveillance frequency could have contributed to differential detection of brain metastasis.

Patients were not required to complete a fixed minimum number of surveillance MRI examinations to remain in the cohort, provided that follow-up information was sufficient to establish their event or censoring status.

### 2.7 Statistical analysis

Statistical analyses were performed using SPSS version 26.0 and R software. Continuous variables were summarized as mean ± standard deviation for approximately normally distributed data and as median with interquartile range for skewed distributions. Categorical variables were summarized as frequencies and percentages. Distributional assumptions were evaluated using graphical methods and the Shapiro Wilk test where appropriate.

Baseline characteristics were compared descriptively between patients who developed brain metastasis and those who did not during observed follow-up using the independent-samples t-test or Mann Whitney U test for continuous variables and the chi-square test or Fisher’s exact test for categorical variables, as appropriate. These comparisons were descriptive and were not used as the sole basis for predictor selection.

#### 2.7.1 Primary time-to-event analysis

Because brain metastasis occurred during variable follow-up periods and some patients were right-censored, the primary association analysis used Cox proportional hazards regression rather than logistic regression.

Univariable Cox models were used to describe associations between individual baseline characteristics and time to brain metastasis, with results reported as HRs and 95% CIs. These univariable analyses were descriptive only and were not used as a screening step for multivariable model entry. Candidate predictors were prespecified according to clinical relevance, prior evidence, data completeness, and avoidance of redundancy rather than statistical significance in univariable analyses.

Candidate variables for multivariable prediction modeling were determined before final model fitting on the basis of clinical relevance, previous evidence, data completeness, and avoidance of redundancy rather than by applying a univariable P-value threshold. Given the limited number of brain metastasis events, the number of candidate predictors was deliberately restricted.

Potential multicollinearity among candidate predictors was assessed using correlation analysis and variance inflation factors (VIFs). Because overall clinical stage is constructed from the TNM classification and therefore overlaps substantially with T and N categories, overall clinical stage was excluded *a priori* from the multivariable candidate set when T and N classifications were modeled separately. Among variables retained for model development, all VIFs were <3, indicating no important residual multicollinearity.

The proportional hazards assumption was evaluated using Schoenfeld residuals and complementary graphical assessment. Where evidence of substantial non-proportionality was identified, the affected variable was further examined using appropriate time-dependent or stratified approaches.

#### 2.7.2 Penalized predictor selection and model development

To reduce instability and overfitting associated with conventional stepwise variable selection in the small cohort, predictor reduction was performed using least absolute shrinkage and selection operator (LASSO) penalized Cox regression.

The penalty parameter was determined through cross-validation within the model-development procedure. Predictors retained by the penalized model were subsequently incorporated into a parsimonious Cox proportional hazards model for estimation of clinically interpretable hazard ratios and construction of the exploratory prediction nomogram.

The resulting nomogram estimated individualized 12- and 24-month brain-metastasis-free probabilities rather than an unspecified overall probability of brain metastasis.

Given the small cohort and limited number of brain metastasis events, the dataset was not randomly divided into training and testing subsets, because such splitting would substantially reduce the effective sample size and further destabilize both model development and validation. Bootstrap internal validation using the full cohort was therefore preferred.

#### 2.7.3 Internal validation and model performance

Internal validation was performed using 1,000 bootstrap resamples. To account for optimism introduced during predictor selection and model fitting, the complete model-development procedure, including penalized predictor selection and subsequent model estimation, was repeated within each bootstrap sample.

Model discrimination was quantified using Harrell’s concordance index (C-index). Both the apparent C-index and the bootstrap optimism-corrected C-index were reported.

Time-dependent receiver operating characteristic analysis was additionally performed to estimate model discrimination at the prespecified 12- and 24-month prediction horizons.

Calibration was evaluated by comparing predicted and observed brain-metastasis-free probabilities at 12 and 24 months. Bootstrap-corrected calibration plots and calibration slope were reported. Prediction error was additionally assessed using the Brier score.

Decision curve analysis was performed at the same prespecified time horizons to explore the potential net benefit of model-based risk stratification across clinically relevant threshold probabilities. Because the model was developed and internally validated within the same small cohort, decision curve findings were interpreted as exploratory and not as evidence supporting specific treatment interventions.

#### 2.7.4 Competing-risk and treatment sensitivity analyses

Because death before brain metastasis prevents subsequent occurrence or detection of the primary outcome, cumulative incidence functions were used to estimate brain metastasis risk while accounting for death as a competing event.

A Fine Gray proportional subdistribution hazards model was used as a sensitivity analysis to assess whether associations observed in the primary Cox analysis remained directionally consistent after accounting for competing mortality. Results were expressed as subdistribution hazard ratios (sHRs) with 95% confidence intervals.

An additional exploratory sensitivity analysis incorporated EGFR-TKI treatment as a time-dependent covariate to examine whether adjustment for treatment exposure materially changed associations between baseline predictors and brain metastasis risk.

#### 2.7.5 Exploratory EGFR subgroup analysis

Given the clinical relevance of EGFR alterations to central nervous system progression, BMFS according to EGFR mutation status was examined using Kaplan Meier curves and the log-rank test. Cumulative incidence functions were additionally examined in the presence of competing mortality.

Analyses according to individual EGFR mutation subtypes, including exon 19 deletion and L858 R mutation, were considered descriptive only because of the limited number of patients and events within individual molecular subgroups. No definitive comparative inference between EGFR mutation subtypes was prespecified.

All statistical tests were two-sided. A P value < 0.05 was considered statistically significant for conventional association analyses. Given the exploratory nature of the prediction model and subgroup analyses, emphasis was placed on effect estimates, confidence intervals, model stability, and internal validation rather than statistical significance alone.

### 2.8 Ethical considerations

The study was conducted in accordance with the Declaration of Helsinki and relevant institutional guidelines and was approved by the Institutional Ethics Committee of The Second People’s Hospital of Liaocheng. Because of the retrospective design and use of routinely collected de-identified clinical data, the requirement for individual informed consent was waived by the ethics committee.

All data were handled confidentially, and personally identifiable information was removed before analysis.

## 3 Results

### 3.1 Cohort construction, follow-up, and brain metastasis events

A total of 94 consecutive patients with pathologically confirmed LUAD diagnosed between January 2018 and December 2024 were screened for eligibility (Figure 1). Twenty-eight patients were excluded because of brain metastasis detected on baseline MRI (n = 17), incomplete baseline clinicopathological or molecular data (n = 6), a concurrent or recent active malignancy (n = 3), or absence of any post-baseline follow-up information (n = 2). The remaining 66 patients constituted the final time-to-event cohort, representing all eligible patients identified from the institutional source population during the study period.

The index date was defined as the date of the baseline contrast-enhanced brain MRI demonstrating no evidence of intracranial metastasis. Patients who subsequently developed brain metastasis within 3 months after the index date were retained as incident events, and patients with short follow-up were retained and censored at their last documented assessment rather than excluded.

During a median follow-up of 27.8 months (IQR, 14.6–43.9 months; range, 1.8–72.0 months), 26 patients developed incident brain metastasis. The median time from the index date to brain metastasis was 15.7 months (IQR, 8.4–27.6 months). Of the 66 patients, 26 developed incident brain metastasis, 9 died before documented brain metastasis, and 31 were alive without documented brain metastasis at their last follow-up. Thus, 40 patients were censored without a BM event in the primary cause-specific Cox analysis, including 9 censored at death and 31 censored at their last documented clinical or imaging assessment. Death before BM was additionally treated as a competing event in the Fine Gray sensitivity analysis.

### 3.2 Baseline clinical and treatment characteristics

The baseline demographic, clinicopathological, molecular, and treatment characteristics of the 66 included patients are summarized in Table 1. The mean age was 58.7 ± 10.1 years, and 37 patients (56.1%) were male. Compared with patients who remained free of brain metastasis during observed follow-up, those who subsequently developed brain metastasis more frequently had T3∼4 disease (61.5% vs. 30.0%), N1∼3 nodal involvement (80.8% vs. 52.5%), EGFR mutation (73.1% vs. 42.5%), and serum CEA >5 ng/mL (65.4% vs. 37.5%).

**TABLE 1:** Baseline demographic, clinicopathological, and molecular characteristics of the study cohort.

Characteristic	Overall cohort (n = 66)	Incident BM (n = 26)	No BM during follow-up (n = 40)	P value
Overall demographic characteristics				
Age, years, mean ± SD	58.7 ± 10.1	​	​	​
Male sex, n (%)	37 (56.1)	​	​	​
Female sex, n (%)	29 (43.9)	​	​	​
T classification, n (%)	​	​	​	0.011
T1∼2	38 (57.6)	10 (38.5)	28 (70.0)	​
T3∼4	28 (42.4)	16 (61.5)	12 (30.0)	​
N classification, n (%)	​	​	​	0.02
N0	24 (36.4)	5 (19.2)	19 (47.5)	​
N1∼3	42 (63.6)	21 (80.8)	21 (52.5)	​
EGFR mutation status, n (%)	​	​	​	0.015
Wild-type	30 (45.5)	7 (26.9)	23 (57.5)	​
Mutant	36 (54.5)	19 (73.1)	17 (42.5)	​
Serum CEA, n (%)	​	​	​	0.027
≤5 ng/mL	34 (51.5)	9 (34.6)	25 (62.5)	​
>5 ng/mL	32 (48.5)	17 (65.4)	15 (37.5)	​

Overall clinical stage was also more advanced among patients who developed brain metastasis. However, because overall stage is derived largely from T and N classifications, clinical stage was used only for descriptive comparison and was not entered simultaneously with T and N stage in multivariable model development.

Regarding systemic treatment during follow-up, 34 of 66 patients (51.5%) received an EGFR-TKI, including 14 (21.2%) treated with osimertinib and 20 (30.3%) treated with first- or second-generation EGFR-TKIs. Chemotherapy was administered to 43 patients (65.2%), immune checkpoint inhibitor therapy to 17 (25.8%), and extracranial radiotherapy to 21 (31.8%). Because EGFR-TKI initiation occurred after the index date in a proportion of patients, EGFR-TKI exposure was analyzed as a time-dependent covariate in sensitivity analysis rather than as a fixed baseline characteristic. The distribution and timing of major systemic treatments are presented in Supplementary Table S1. During follow-up, patients underwent a median of 4 brain MRI examinations (IQR, 3–6), with a median interval of 5.1 months (IQR, 3.8–6.4 months) between surveillance examinations. The median surveillance interval was 4.8 months (IQR, 3.6–6.1 months) among patients who subsequently developed BM and 5.3 months (IQR, 4.0–6.6 months) among those who did not, with no clear evidence of a major between-group difference (P = 0.41).

### 3.3 Time-to-event analysis of brain metastasis

Because follow-up duration varied substantially among patients and some observations were right-censored, incident brain metastasis was analyzed as a time-to-event outcome using Cox proportional hazards regression rather than as a binary BM versus non-BM endpoint. BMFS was measured from the baseline negative brain MRI (index date) to the first radiologically confirmed BM, with patients free of BM censored at death or their last documented follow-up, as appropriate.

In univariable Cox regression analyses, T3∼4 classification, N1∼3 nodal involvement, EGFR mutation, and CEA >5 ng/mL were associated with an increased hazard of subsequent brain metastasis. T3∼4 disease was associated with an HR of 2.63 (95% CI, 1.20–5.76; P = 0.016), N1∼3 involvement with an HR of 2.71 (95% CI, 1.12–6.54; P = 0.027), EGFR mutation with an HR of 2.58 (95% CI, 1.12–5.94; P = 0.026), and CEA >5 ng/mL with an HR of 2.12 (95% CI, 0.98–4.60; P = 0.056). Age <60 years showed a weaker association (HR, 1.54; 95% CI, 0.71–3.34; P = 0.274). Univariable and multivariable Cox regression results are summarized in Table 2.

**TABLE 2:** Univariable and multivariable cox regression analyses for incident brain metastasis.

Variable	Univariable HR (95% CI)	P value	Multivariable HR (95% CI)	P value
Age <60 years	1.54 (0.71–3.34)	0.274	—	—
T3–4 classification	2.63 (1.20–5.76)	0.016	2.31 (1.02–5.22)	0.045
N1–3 involvement	2.71 (1.12–6.54)	0.027	2.42 (1.00–5.87)	0.049
EGFR mutation	2.58 (1.12–5.94)	0.026	2.49 (1.06–5.85)	0.036
CEA >5 ng/mL	2.12 (0.98–4.60)	0.056	2.04 (0.93–4.47)	0.075

Univariable Cox analyses were performed for descriptive association assessment only and were not used to determine entry into the multivariable model. Candidate predictors were prespecified on the basis of clinical relevance, prior evidence, data completeness, and avoidance of redundancy. Given the limited number of BM events, the candidate set was deliberately restricted before penalized model fitting. Because overall clinical stage is derived from the TNM classification and overlaps substantially with T and N categories, it was excluded from the multivariable candidate set to avoid structural redundancy. Collinearity assessment of the retained predictors showed no important residual multicollinearity, with all VIFs <3.

LASSO-penalized Cox regression retained four variables T classification, N classification, EGFR mutation status, and serum CEA for the exploratory prediction model (Supplementary Figure S1). In the corresponding parsimonious Cox model, T3∼4 classification (HR, 2.31; 95% CI, 1.02–5.22), N1∼3 involvement (HR, 2.42; 95% CI, 1.00–5.87), and EGFR mutation (HR, 2.49; 95% CI, 1.06–5.85) showed associations with higher BM hazard, whereas the estimate for CEA >5 ng/mL was less precise (HR, 2.04; 95% CI, 0.93–4.47). Given the limited number of events, these coefficients and effect estimates were considered exploratory.

Kaplan-Meier analysis demonstrated shorter BMFS among patients with EGFR-mutant tumors than among those with EGFR-wild-type tumors (median BMFS, 22.4 months vs. not reached; log-rank P = 0.009; Figure 2). The estimated brain-metastasis-free survival probabilities for the overall cohort were 82.1% at 12 months and 66.4% at 24 months.

**FIGURE 2:**
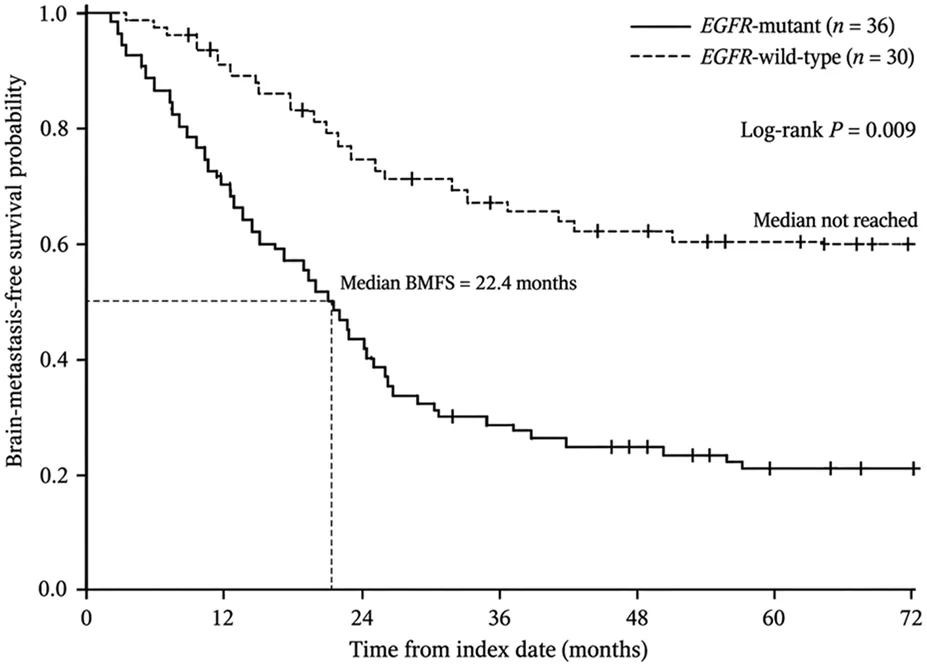
Kaplan Meier curves for brain-metastasis-free survival according to EGFR mutation status. Kaplan Meier curves comparing brain-metastasis-free survival between patients with EGFR-mutant and EGFR-wild-type lung adenocarcinoma. Patients with EGFR-mutant tumors showed shorter BMFS than those with EGFR-wild-type tumors (median BMFS, 22.4 months vs. not reached; log-rank P = 0.009). Tick marks indicate censored observations. EGFR, epidermal growth factor receptor; BMFS, brain-metastasis-free survival.

### 3.4 Exploratory time-to-event prediction model and internal validation

An exploratory Cox-based nomogram was constructed using T stage, N stage, EGFR mutation status, and serum CEA level to estimate individualized 12- and 24-month brain-metastasis-free probabilities (Figure 3). Model complexity was intentionally restricted because of the limited number of outcome events.

**FIGURE 3:**
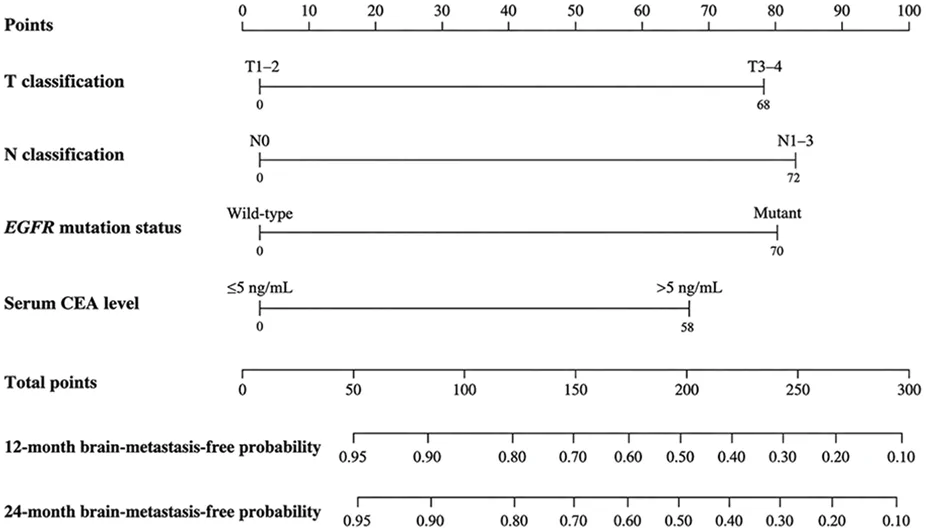
Cox-based nomogram for predicting 12- and 24-month brain-metastasis-free probabilities in patients with lung adenocarcinoma. The nomogram was constructed using T classification, N classification, EGFR mutation status, and serum CEA level to estimate individualized 12- and 24-month brain-metastasis-free probabilities. Each predictor is assigned a point value, and the total score corresponds to the estimated probability of remaining free from brain metastasis at the specified time points. EGFR, epidermal growth factor receptor; CEA, carcinoembryonic antigen.

The apparent Harrell C-index of the model was 0.781. Bootstrap internal validation with 1,000 resamples yielded an optimism-corrected C-index of 0.742, corresponding to an estimated optimism of 0.039. Time-dependent ROC analysis showed an AUC of 0.773 (95% CI, 0.665–0.866) at 12 months and 0.758 (95% CI, 0.650–0.850) at 24 months (Figure 4A).

**FIGURE 4:**
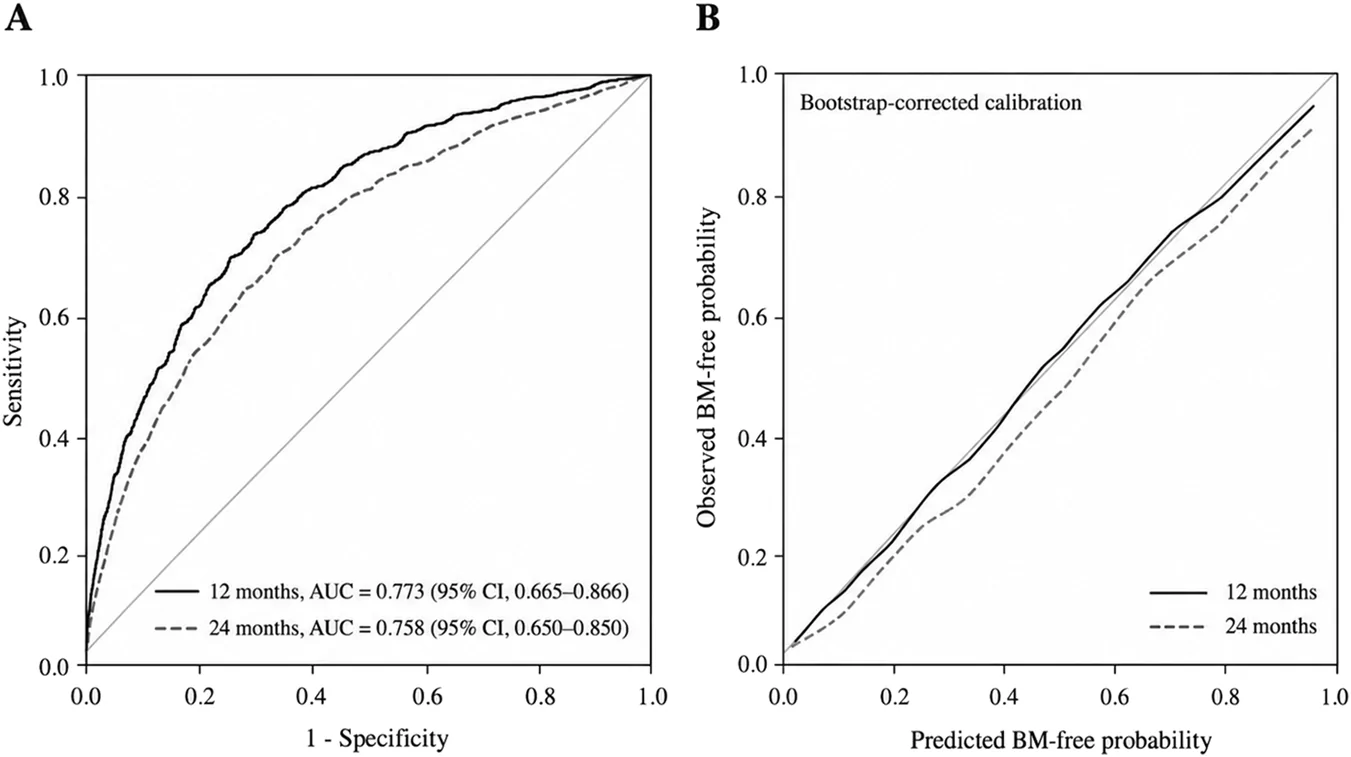
Internal validation of the exploratory Cox-based prediction model. **(A)** Time-dependent receiver operating characteristic curves for prediction of brain-metastasis-free probability at 12 and 24 months. **(B)** Bootstrap-corrected calibration curves comparing predicted and observed brain-metastasis-free probabilities at 12 and 24 months. AUC, area under the curve.

Bootstrap-corrected calibration curves demonstrated reasonable agreement between predicted and observed brain-metastasis-free probabilities at both 12 and 24 months (Figure 4B). The optimism-corrected calibration slope was 0.87, indicating modest residual overfitting relative to the ideal slope of 1.00. The 24-month Brier score was 0.164. Taken together, the corrected discrimination and calibration results indicated moderate internal model performance, although some optimism remained and external validity was not established.

### 3.5 Competing mortality and sensitivity analyses

Nine patients died before documented brain metastasis and were treated as competing events in a Fine Gray sensitivity analysis. The cumulative incidence of brain metastasis was 16.7% at 12 months and 30.8% at 24 months.

The competing-risk analysis showed results broadly consistent with those of the primary Cox model. T3∼4 classification was associated with an sHR of 2.18 (95% CI, 1.01–4.73), N1∼3 involvement with an sHR of 2.27 (95% CI, 1.01–5.11), and EGFR mutation with an sHR of 2.36 (95% CI, 1.06–5.27). The association for CEA >5 ng/mL was directionally similar but less precise (sHR, 1.88; 95% CI, 0.88–4.03). Results of the competing-risk and treatment-adjusted sensitivity analyses are summarized in Table 3.

**TABLE 3:** Competing-risk and treatment-adjusted sensitivity analyses for incident brain metastasis.

Variable	Fine gray sHR (95% CI)	Time dependent EGFR-TKI-adjusted cox HR (95% CI)
T3–4 classification	2.18 (1.01–4.73)	2.18 (0.96–4.95)
N1–3 involvement	2.27 (1.01–5.11)	2.27 (0.93–5.54)
EGFR mutation	2.36 (1.06–5.27)	2.16 (0.88–5.29)
CEA >5 ng/mL	1.88 (0.88–4.03)	1.89 (0.85–4.20)
Time-dependent EGFR-TKI exposure	—	0.76 (0.33–1.75)

In an additional exploratory Cox sensitivity analysis incorporating EGFR-TKI exposure as a time-dependent covariate, the associations of T3–4 classification (HR, 2.18; 95% CI, 0.96–4.95), N1–3 involvement (HR, 2.27; 95% CI, 0.93–5.54), and EGFR mutation (HR, 2.16; 95% CI, 0.88–5.29) with brain metastasis remained directionally consistent with the primary analysis, although the effect estimates were attenuated. The corresponding estimate for CEA >5 ng/mL was HR 1.89 (95% CI, 0.85–4.20). Time-dependent EGFR-TKI exposure was associated with a lower hazard of brain metastasis (HR, 0.76; 95% CI, 0.33–1.75), although the confidence interval was wide and included the null. Because treatment allocation was non-random, treatment patterns changed over the 2018–2024 study period, and only 26 BM events were available, these treatment-adjusted findings were considered exploratory and calendar period was not incorporated as an additional predictor in the primary model.

### 3.6 Exploratory analysis according to EGFR mutation status

Among 36 patients with EGFR-mutant tumors, 19 developed brain metastasis during follow-up, compared with 7 of 30 patients with EGFR-wild-type tumors. The cumulative incidence of brain metastasis was therefore higher in the EGFR-mutant group, consistent with the primary time-to-event analysis.

Among EGFR-mutant patients, 17 had an exon 19 deletion and 16 had an L858 R mutation, while 3 had other uncommon EGFR variants. Brain metastasis occurred in 9 of 17 patients with exon 19 deletion and 9 of 16 patients with L858 R mutation. Because of the small number of patientsandevents within individual molecular subgroups, comparisons between EGFR mutationsubtypes were treated as descriptive only, and no formal inference regarding differential metastatic risk was made. The distribution of EGFR mutation subtypes and corresponding BM events is summarized in Supplementary Table S2.

## 4 Discussion

In this retrospective cohort study of Chinese patients with LUAD who had no evidence of brain metastasis at baseline, we evaluated subsequent brain metastasis as a time-to-event outcome and developed an exploratory prediction model incorporating routinely available clinicopathological and molecular characteristics. Advanced T classification, regional nodal involvement, EGFR mutation, and elevated serum CEA were associated with a higher subsequent hazard of brain metastasis. A parsimonious Cox-based model incorporating these variables demonstrated moderate discrimination and acceptable internal calibration after bootstrap correction. Importantly, because of the limited sample size and number of brain metastasis events, the present model should be regarded as exploratory rather than as a fully validated clinical decision tool. Its principal value lies in hypothesis generation and preliminary risk stratification, pending confirmation in larger independent cohorts.

The association between more advanced locoregional disease and subsequent brain metastasis is consistent with previous evidence. Higher T classification reflects greater primary tumor burden and, in many cases, more extensive local invasion, whereas nodal involvement indicates established lymphatic dissemination and a biologically more aggressive disease phenotype (Wang et al., 2021). Large population-based analyses have similarly reported associations between advanced T or N classification and an increased probability of brain metastasis in NSCLC (Huang et al., 2020). In the present study, both T3∼4 disease and N1∼3 involvement remained associated with shorter brain-metastasis-free survival in time-to-event analyses. These findings are biologically plausible because increasing tumor burden and nodal spread may provide greater opportunity for hematogenous dissemination and distant organ colonization. However, because overall clinical stage is largely derived from T and N categories, we did not enter overall stage simultaneously with both components in the final model, thereby reducing redundancy and potential collinearity.

The association between EGFR mutation and subsequent brain metastasis also warrants careful interpretation. Previous studies and meta-analyses have reported a higher incidence of brain metastasis among patients with EGFR-mutant NSCLC than among those with EGFR-wild-type disease (Shin et al., 2014). Several mechanisms may contribute to this relationship. EGFR signaling can influence tumor cell proliferation, adhesion, migration, and interactions with the blood brain barrier, potentially facilitating neurotropic dissemination (Er et al., 2017). In addition, the clinical course of EGFR-mutant LUAD has been substantially altered by tyrosine kinase inhibitor (TKI) therapy, which may influence both survival duration and the pattern of central nervous system progression. Earlier-generation EGFR-TKIs had relatively limited central nervous system penetration, whereas later-generation agents such as osimertinib achieve greater intracranial activity. Therefore, the observed relationship between EGFR mutation and brain metastasis should not be interpreted as a purely intrinsic biological effect independent of treatment exposure. Previous studies, including analyses of surgically treated pulmonary adenocarcinoma, have nevertheless identified EGFR mutation as a predictor of subsequent brain metastasis (Suda et al., 2021). Our findings are consistent with this broader literature, although treatment heterogeneity and treatment timing remain important potential confounders.

In the present study, treatment effects were examined more explicitly than in the original analysis. In particular, EGFR-TKI use was considered in sensitivity analyses because treatment initiation frequently occurred after cohort entry and therefore could not be appropriately handled as a simple fixed baseline characteristic. Modeling treatment exposure as time dependent reduces, although does not eliminate, bias related to treatment timing. The direction of the principal associations remained broadly similar after accounting for EGFR-TKI exposure. Nevertheless, these analyses should be interpreted cautiously because treatment allocation was not randomized and was influenced by mutation status, disease stage, calendar period, physician preference, and overall clinical condition. Consequently, the present study cannot distinguish whether the observed association between EGFR mutation and brain metastasis reflects tumor biology, treatment exposure, prolonged survival, or a combination of these factors.

Elevated serum CEA was also associated with a higher risk of subsequent brain metastasis, although its effect estimate was less precise than those of some other predictors. CEA is a widely available biomarker in routine LUAD management and has previously been associated with tumor burden, metastatic potential, and adverse outcomes (Buccheri and Ferrigno, 2001). Biologically, CEA is involved in cell adhesion and tumor host interactions and may facilitate metastatic colonization through effects on circulating tumor cell survival and tissue-specific adhesion (Beauchemin and Arabzadeh, 2013). Previous LUAD prediction studies have incorporated CEA into multivariable risk models for brain metastasis, supporting its potential value as a readily measurable component of multimarker risk assessment (Zuo et al., 2021). However, because serum CEA may be influenced by disease burden, smoking status, and other clinical factors, it should not be regarded as a brain-specific biomarker. Its contribution is therefore better interpreted in combination with anatomical and molecular variables rather than in isolation.

An important methodological feature of the revised analysis is that brain metastasis was treated as a time-to-event outcome rather than as a simple binary endpoint. This distinction is clinically relevant because patients had heterogeneous follow-up durations, and some were censored before experiencing brain metastasis. Logistic regression would treat a patient followed for only a few months without brain metastasis similarly to one followed for several years without brain metastasis, thereby ignoring unequal observation time. By using Cox regression, the present analysis incorporated follow-up duration and right censoring and allowed prediction of brain-metastasis-free probability at clinically interpretable time horizons. In addition, patients with short follow-up were censored rather than excluded, and patients who developed brain metastasis shortly after a negative baseline MRI were retained as incident events. These changes reduced the risk of selection and survivor bias associated with post-baseline exclusion criteria.

Death before brain metastasis represents another important consideration in this population, particularly because many patients had advanced-stage disease. In the primary Cox analysis, deaths before brain metastasis were censored; however, this approach may overestimate the cumulative incidence of brain metastasis when mortality is substantial because death precludes subsequent observation of the event. We therefore performed a competing-risk sensitivity analysis using the Fine Gray framework. The direction of the main associations was generally preserved after accounting for competing mortality, providing some reassurance regarding the robustness of the findings. Nevertheless, the small number of deaths and brain metastasis events limits the precision of these estimates, and larger cohorts are required for more reliable competing-risk modeling.

The prediction model itself should also be interpreted conservatively. Previous studies have developed nomograms and other multivariable models for predicting brain metastasis in NSCLC or LUAD using combinations of stage, nodal status, serum markers, and molecular characteristics (Callegaro et al., 2016; An et al., 2018). Accordingly, the primary contribution of the present study is not the identification of entirely novel individual predictors. Rather, the study evaluates these factors within a time-to-event framework while explicitly addressing censoring, competing mortality, model complexity, and internal optimism. Given the limited number of outcome events, conventional univariable screening followed by backward stepwise selection would have created a substantial risk of model instability and exaggerated apparent performance. We therefore restricted the number of candidate predictors, used penalized regression for variable reduction, and repeated the full model-development process within bootstrap resampling. The difference between apparent and optimism-corrected performance further underscores why internal validation is essential in small prediction studies.

The internally validated model showed moderate discriminatory ability and reasonable calibration at the prespecified prediction horizons. These findings suggest that routinely available variables may provide useful information for stratifying patients according to relative brain metastasis risk. However, internal validation does not establish transportability to other institutions or patient populations. In particular, the present cohort was derived from a single Chinese center, and molecular epidemiology, treatment patterns, imaging surveillance, and access to CNS-active therapies may differ across regions. The model should therefore not be used at present to determine treatment selection, prophylactic cranial irradiation, or other preventive interventions. Rather, it may serve as a basis for further evaluation of risk-adapted surveillance strategies in prospective or externally validated studies.

The clinical implications of the present findings should therefore be considered primarily in the context of surveillance and research rather than direct therapeutic intervention. Patients with combinations of advanced T or N classification, EGFR mutation, and elevated CEA may represent a subgroup with a higher observed risk of subsequent brain metastasis. Whether such patients benefit from more frequent brain MRI, earlier CNS-focused assessment, or modification of systemic therapy cannot be established from this retrospective observational study. Current clinical guidelines recommend brain imaging for initial staging in selected patients with NSCLC, but the optimal intensity and frequency of longitudinal CNS surveillance remain incompletely defined. Future studies should prospectively evaluate whether risk-stratified imaging strategies improve clinically relevant outcomes without producing excessive imaging burden or unnecessary intervention.

Several limitations should be acknowledged. First, this was a retrospective single-center study with a small cohort and a limited number of brain metastasis events. Despite model simplification, penalization, and bootstrap correction, residual overfitting remains possible, and coefficient estimates may be unstable. Accordingly, the nomogram should be regarded as exploratory. Second, although the revised analysis accounted for unequal follow-up time and right censoring, surveillance schedules were not completely uniform. Brain MRI was performed according to routine clinical practice and symptom-triggered evaluation, which may have introduced detection bias if imaging intensity differed between patient groups. Third, treatment regimens were heterogeneous and evolved substantially between 2018 and 2024, particularly with the increasing use of third-generation EGFR-TKIs and immunotherapy. Although EGFR-TKI exposure was considered in sensitivity analyses, residual confounding by treatment indication, timing, and treatment era cannot be excluded. Fourth, death before brain metastasis was a competing event. Although competing-risk analyses were performed, the relatively small number of events limited statistical precision. Fifth, molecular characterization was not uniform across all patients because testing platforms and gene panels changed during the study period. Potentially relevant alterations, including TP53, KRAS, copy number changes, and other genomic features, were therefore not systematically incorporated into the model. Sixth, no external validation cohort was available. Independent validation in larger multicenter populations is required before any clinical implementation. Finally, the study did not evaluate whether risk-adapted surveillance or treatment modification based on the model improves survival, neurological outcomes, quality of life, or cost-effectiveness.

In conclusion, advanced T classification, nodal involvement, EGFR mutation, and elevated serum CEA were associated with an increased time-dependent risk of subsequent brain metastasis in this retrospective LUAD cohort. An exploratory Cox-based model integrating these routinely available factors demonstrated moderate internally validated discrimination and calibration. These findings support further investigation of individualized brain metastasis risk stratification but do not establish a basis for direct treatment modification. Larger prospective and multicenter studies with external validation, standardized imaging surveillance, detailed treatment exposure, and competing-risk assessment are needed to determine whether this approach can contribute meaningfully to clinical management.

## Data Availability

The original contributions presented in the study are included in the article/Supplementary Material, further inquiries can be directed to the corresponding author.

## Supplementary material

SUPPLEMENTARY FIGURE S1 LASSO-penalized Cox regression for predictor selection. **(A)** LASSO coefficient profiles of candidate predictors across different values of the penalty parameter (λ). **(B)** Ten-fold cross-validation curve used to determine the optimal λ. The selected penalty parameter retained four predictors for the exploratory Cox model: T classification, N classification, EGFR mutation status, and serum CEA level. LASSO, least absolute shrinkage and selection operator; EGFR, epidermal growth factor receptor; CEA, carcinoembryonic antigen.

SUPPLEMENTARY TABLE S1 Systemic Treatment Exposure and Brain MRI Surveillance During Follow-up.

SUPPLEMENTARY TABLE S2 Descriptive Distribution of EGFR Mutation Subtypes and Brain Metastasis Events.
