# Supplementary material for: Risk factors and time-to-event prediction of brain metastasis in patients with lung adenocarcinoma: a retrospective cohort study
Source: Front Genet. 2026 Oct 28;17:1931724. doi: 10.3389/fgene.2026.1931724 (PMC13750748; doi:10.3389/fgene.2026.1931724)
Supplement: Supplementary file 1 [file Table1.doc]

**Supplementary Table S1. Systemic Treatment Exposure and Brain MRI Surveillance During Follow-up**

| **Characteristic** | **Overall cohort (n=66)** |
| --- | --- |
| **Systemic treatment exposure, n (%)** |  |
| Any EGFR-TKI | 34 (51.5) |
| Osimertinib | 14 (21.2) |
| First- or second-generation EGFR-TKI | 20 (30.3) |
| Chemotherapy | 43 (65.2) |
| Immune checkpoint inhibitor therapy | 17 (25.8) |
| Extracranial radiotherapy | 21 (31.8) |
| **Treatment-timing analysis** |  |
| EGFR-TKI exposure modeled as a time-dependent covariate | Yes |
| Exposure status before EGFR-TKI initiation | Unexposed |
| Exposure status after EGFR-TKI initiation | Exposed |
| **Brain MRI surveillance** |  |
| Number of follow-up brain MRI examinations, median (IQR) | 4 (3–6) |
| Interval between surveillance MRI examinations, months, median (IQR) | 5.1 (3.8–6.4) |
| Surveillance interval in patients who developed BM, months, median (IQR) | 4.8 (3.6–6.1) |
| Surveillance interval in patients without BM, months, median (IQR) | 5.3 (4.0–6.6) |
| Between-group comparison of surveillance interval | P=0.41 |
