# Supplementary material for: Risk factors and time-to-event prediction of brain metastasis in patients with lung adenocarcinoma: a retrospective cohort study
Source: Front Genet. 2026 Oct 28;17:1931724. doi: 10.3389/fgene.2026.1931724 (PMC13750748; doi:10.3389/fgene.2026.1931724)
Supplement: Supplementary file 2 [file Table2.doc]

**Supplementary Table S2. Descriptive Distribution of EGFR Mutation Subtypes and Brain Metastasis Events**

| **EGFR status/subtype** | **Total, n** | **BM events, n (%)** |
| --- | --- | --- |
| EGFR wild-type | 30 | 7 (23.3) |
| EGFR mutant, overall | 36 | 19 (52.8) |
| Exon 19 deletion | 17 | 9 (52.9) |
| L858R mutation | 16 | 9 (56.3) |
| Other uncommon EGFR variants | 3 | 1 (33.3) |
